# Levers to Improve Antibiotic Treatment of Lambs via Drinking Water in Sheep Fattening Houses: The Example of the Sulfadimethoxine/Trimethoprim Combination

**DOI:** 10.3390/antibiotics9090561

**Published:** 2020-08-31

**Authors:** Aude A. Ferran, Marlène Z. Lacroix, Alain Bousquet-Mélou, Ivain Duhil, Béatrice B. Roques

**Affiliations:** INTHERES, Université de Toulouse, INRAE, ENVT, 31300 Toulouse, France ; aude.ferran@envt.fr (A.A.F.); marlene.lacroix@envt.fr (M.Z.L.); alain.bousquet-melou@envt.fr (A.B.-M.); duhilivain@gmail.com (I.D.)

**Keywords:** drinking water, antibiotic, lamb, trimethoprim, sulfonamides, pharmacokinetics, metaphylaxis

## Abstract

To limit the spread of bacterial diseases in sheep fattening houses, antibiotics are often administered collectively. Collective treatments can be delivered by drinking water but data on the drug’s solubility in water or on plasma exposure of the animals are lacking. We first assessed the solubility of products containing sulfadimethoxine (SDM), associated or not with trimethoprim (TMP), in different waters. We then compared in lambs the SDM and TMP pharmacokinetic profiles after individual intravenous (IV) and oral administrations of SDM-TMP in experimental settings (n = 8) and after a collective treatment by drinking water with SDM-TMP or SDM alone in a sheep fattening house (n = 100 for each treatment). The individual water consumption during the collective treatments was also monitored to characterize the ingestion variability. We showed that TMP had a short terminal half-life and very low oral bioavailability, demonstrating that it would be unable to potentiate SDM by oral route. Conversely, SDM had a long terminal half-life of 18 h and excellent oral bioavailability. However, delivery by drinking water resulted in a very high interindividual variability of SDM plasma concentrations, meaning that although disease spread could be controlled at the group level, some individuals would inevitably be under- or over-exposed to the antibiotic.

## 1. Introduction

The management of pulmonary diseases in sheep fattening houses often relies on the administration of antibiotics to sick animals but also to contaminated ones to prevent the spread of the infection. Indeed, it has been shown that early/metaphylactic antibiotic treatments are more efficacious than curative treatments administered only to clinically sick animals [1,2]. Due to the high density of animals in fattening houses, tens or hundreds of animals need to be treated during the epidemic stage of disease, which precludes individual administrations of the antibiotics by intramuscular or subcutaneous route.

In such cases, a collective oral treatment via the feed or drinking water is required even if, in the context of a prudent use of antimicrobials in veterinary medicine, mass medication is of major concern [3]. In the recent EMA (European Medicines Agency) categorization of antibiotics for prudent and responsible use in animals, oral group medications were classified as high risk for resistance selection, the risk being greater for medicated food than for drinking water [3]. Indeed, delivery of a drug in drinking water is more flexible than in feed. Medicated drinking water can be instantaneously prepared and the doses and volumes easily modulated every day [4]. However, most of the oral formulations available for sheep were developed for direct administration as a bolus in the animal’s mouth and no or very few data are available on the drug’s solubility and stability in water. Moreover, the drinking water in fattening houses can come from the municipal water supply or from underground water extracted from a well and can have very different chemical properties ranging from acidic to basic and different hardnesses which can impair drug’s solubility. In a recent study by Vandael et al. [5], 33 out of 52 pig farmers reported some practical problems, such as solubility issues and precipitation, with drinking water medication.

In addition to solubility and stability issues in the pipelines, group medication exhibits additional variability associated with individual drinking behaviors, which can lead to overexposure or underexposure of animals to the antibiotic. In pigs, Soraci et al. [6] showed that the plasma exposure of animals to fosfomycin varied considerably between pigs after administration in the feed or drinking water and that this interindividual variability, lower for drinking water, could be partly explained by the social rank of the animal. However, apart from ensuring adequate access of the animals to the water, interindividual variability is probably very difficult to manage in the field. Therefore, any factor that might contribute to poor plasma exposure to antibiotics, such as product solubility or dosage regimen, needs to be carefully optimized to limit treatment failures and the selection of antibiotic resistance.

A combination of sulfonamides and diaminopyrimidines has long been used in veterinary medicine to manage bacterial and protozoal infections and is frequently employed to control respiratory infections in cattle and sheep [7]. These antibiotics have been classified as low risk by EMA [3] and are considered suitable for first-line treatments in veterinary medicine. Sulfonamides and diaminopyrimidines are considered as primarily bacteriostatic but become synergistic and bactericidal when used in combination by inhibiting different steps of tetrahydrofolate biosynthesis [8]. Many sulfonamides (sulfadiazine, sulfadimethoxine, sulfamethoxypyridazine, sulfamethoxazole, sulfadoxine or sulfadimidine) are available in combination with trimethoprim (TMP) in veterinary medicine while only sulfamethoxazole is registered in combination with TMP in human medicine. The terminal half-life of TMP is less than one hour in sheep [9,10] whereas very different half-lives have been reported for the various sulfonamides. Sulfadiazine or sulfathiazole with half-lives in sheep of 4 h [10] and 1.1 h [11] respectively are considered as short-acting sulfonamides while sulfadimethoxine (SDM) with a half-life of 12.5 h in cattle [12] is a long-acting one. Whatever the pharmacokinetic properties of drugs, most of the formulations registered in animals contain a 5:1 ratio for sulfonamides and TMP, which was originally extrapolated from human medicine even though data on the relevance of this ratio in veterinary species are very sparse. The main advantage of a long half-life is that fluctuations in the concentrations during the dosing interval are low, requiring less frequent administrations, while the drawback can be the delay for plasma concentrations to reach steady-state [13]. The bioavailability after administration by oral route can also differ, with bioavailabilities ranging from 12 to 68% in dwarf goats for some sulfonamides [14] and from 44 to 84% in goats for others [15]. In sheep, the oral bioavailabilities reported for sulfadiazine and sulfamethazine are around 69% [10] and 58% [16], respectively. No value for the bioavailability of TMP after oral route in ruminants were found but Shoaf et al. [17] reported very low concentrations after oral treatment in 12-week old calves.

In this study, we investigated the exposure of sheep to SDM and TMP after administration of the SDM-TMP combination or SDM alone at the recommended doses in the drinking water. We first compared the oral and intravenous pharmacokinetic profiles of the drugs in experimental settings and then assessed, in field conditions, the drug concentrations in the drinking water and in lamb plasma after delivery of the drug via the drinking water. In vitro solubility assays were performed to identify and investigate the factors limiting adequate drug exposure, by comparing the oral pharmacokinetic profiles in pre-ruminating and ruminating lambs, assessing the drug concentrations in the troughs, and by monitoring individual drinking behaviors.

## 2. Results

### 2.1. Solubility in Water of Formulations Containing the SDM-TMP Combination or SDM Alone

One of the two tested SDM-TMP formulations, Trisulmix^®^ Powder, was not soluble in water after 2 h at RT or at +30 °C. Dissolution of this formulation was facilitated by adding the organic diluent (Trisulmix^®^ Powder:Super Diluant Virbac^®^, 3:1), but as soon as water was added, the product precipitated whatever the pH or hardness of the water.

The same SDM-TMP combination in liquid form, Trisulmix^®^ Liquid, was more soluble than the powder form as, without the organic diluent, only a very slight deposit was observed at RT and at +4 °C for almost all the pH and hardness conditions. However, a heavy precipitate was observed for hard water (50 °f) at pH 5.5. Addition of the organic diluent (Trisulmix^®^ Liquid:Super Diluant Virbac^®^ 2:1) led to excellent solubility after 2 h at RT or +4 °C whatever the pH and hardness conditions.

The SDM formulation tested, Emericid^®^ Sulfadimethoxine, was soluble in water at RT and +4 °C even if the solution appeared cloudier as pH and—especially—water hardness were increased.

Thus, the Trisulmix^®^ Liquid formulation mixed with the organic diluent was used both to determine the pharmacokinetic parameters of SDM and TMP in experimental settings and in the first field experiment. The second field experiment in the sheep fattening house was carried out with Emericid^®^ Sulfadimethoxine.

### 2.2. Pharmacokinetic Parameters of SDM and TMP in Experimental Settings

**Figure 1:**
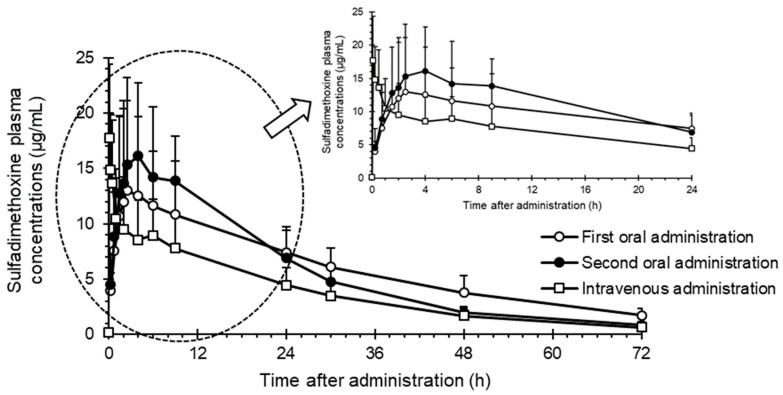
Sulfadimethoxine (SDM) plasma concentrations (mean ± SD) after single administrations of a SDM-trimethoprim (TMP) combination orally (Trisulmix^®^ Liquid, 24.7 mg/kg SDM + 5.3 mg/kg TMP, open circles = first oral administration, closed circles = second oral administration) or intravenously (Trisulmix^®^ Injectable, 24.7 mg/kg SDM + 5.3 mg/kg TMP, open squares) to eight lambs.

Pharmacokinetic profiles of SDM and TMP were obtained for 8 lambs after successive administrations of SDM-TMP by oral, intravenous, and again by oral routes. The SDM plasma concentration profiles are shown in Figure 1. The SDM concentrations remained above the LOQ of the assay (0.25 µg/mL) until 72 h after SDM-TMP administration, both by oral and intravenous routes. The pharmacokinetic parameters of SDM obtained after non-compartmental analysis are presented in Table 1. For the 8 sheep tested, the peak SDM plasma concentration (C_max_) ranged from 6.91 to 35.62 µg/mL for the oral routes. The terminal half-lives (t_1/2_) ranged from 14.00 to 31.65 h and from 13.19 to 19.34 h for the oral routes and the intravenous route, respectively. Only the terminal half-life differed significantly between the first and second oral routes (*p* < 0.05) while the AUC_0-inf_ (Area Under Curve from time 0 extrapolated to infinite time), the apparent clearance (Cl/F), the C_max_, the time at which this concentration was reached (T_max_), the apparent volume of distribution at steady-state (Vss/F) and the bioavailability of SDM were not significantly different (*p* > 0.05) suggesting that the effect of lamb age and ruminal status on SDM pharmacokinetic parameters was negligible.

**Figure 2:**
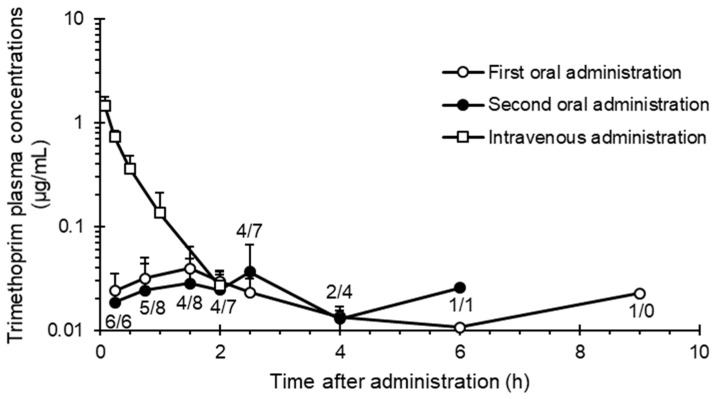
TMP plasma concentrations (mean ± SD) after single administrations of a SDM-TMP combination orally (Trisulmix^®^ Liquid, 24.7 mg/kg SDM + 5.3 mg/kg TMP, open circles = first oral administration, closed circles = second oral administration) or intravenously (Trisulmix^®^ Injectable, 24.7 mg/kg SDM + 5.3 mg/kg TMP, open squares) to eight lambs. For the intravenous administrations, concentrations were above the LOQ (Limit of Quantification) of 0.01 µg/mL from 0.08 to 2 h for all lambs and below the LOQ for all the other sampling times. For the oral administrations, the number of samples with concentrations above the LOQ are indicated on the graph (x/x = numbers of samples > LOQ for the first oral route/numbers of samples > LOQ for the second oral route).

The TMP plasma concentrations profiles are shown in Figure 2. After the intravenous administration, the TMP plasma concentrations remained above the LOQ of 0.01 µg/mL only up to 2 h. The pharmacokinetic parameters of TMP obtained after non-compartmental analysis of the intravenous profiles are presented in Table 2. The terminal half-life ranged from 0.32 to 1.03 h. After the oral administrations, because the TMP plasma concentrations were below the LOQ in 76 samples out of 144 during the first 24 h, the pharmacokinetic parameters could not be precisely estimated. For two animals out of eight, the concentrations were below the LOQ for all sampling times after the first oral administration. The highest concentration obtained in one sheep after oral administration was 0.084 µg/mL.

### 2.3. Individual Water Consumption in Fattening Houses

**Figure 3:**
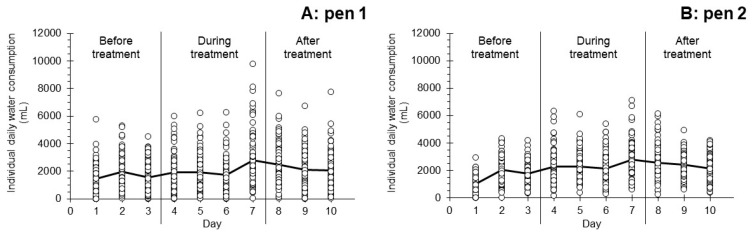
Individual daily water consumption of 96 lambs in pen 1 (4 lambs were not detected at the drinking troughs) and 98 lambs in pen 2 (1 lamb was not detected and 1 lamb died during the experiment), 3 days before, 4 days during and 3 days after the administration of a SDM-TMP combination (Trisulmix^®^ Liquid, 37.4 mg/kg/24 h SDM + 8.0 mg/kg/24 h TMP) in the drinking water. Each circle corresponds to the daily water consumption of one lamb. The solid line links the average daily water consumption.

**Figure 4:**
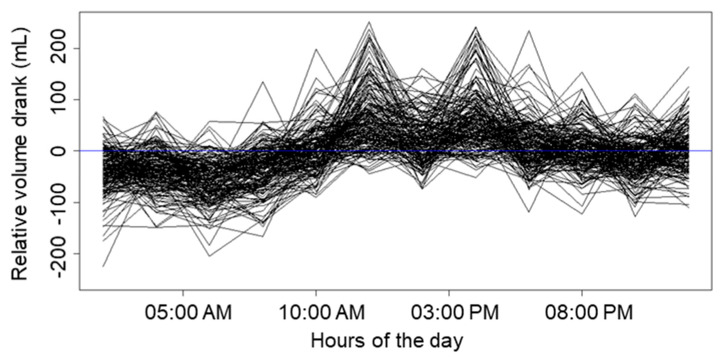
Individual circadian rhythms for water consumption of all lambs in pens 1 and 2 for the first field experiment.

For the first field experiment, the individual daily water consumption over the 10 days of recordings for animals weighing around 24 kg on average (min: 15.2 kg, max: 35.7 kg) ranged from 0 to 9.8 L and from 0 to 7.1 L for pen 1 (without blood samplings) and pen 2 (with blood samplings), respectively (Figure 3). The average volumes consumed over the period were 2.0 ± 1.3 and 2.1 ± 1.1 L/d for pen 1 and pen 2, respectively, which was slightly lower than the expected volume of 2.5 L/d. Individual daily water consumption varied considerably from one lamb to another for the same day of treatment and from one day to another for the same lamb. The average daily water consumption did not decrease following addition of the SDM-TMP combination to the drinking water, suggesting that palatability of the treatment was not a problem. In the same way as for individual daily water consumption, the circadian cycle was different for each lamb (Figure 4). However, a trend was detected for all lambs with a higher water intake between 10 a.m. and 8 p.m. and a lower one overnight. Two consumption peaks could be observed at around 12 p.m. and 4 p.m.

### 2.4. Drug Concentrations in Drinking Troughs

#### 2.4.1. First Field Experiment with the SDM-TMP Combination

**Scheme 1:**
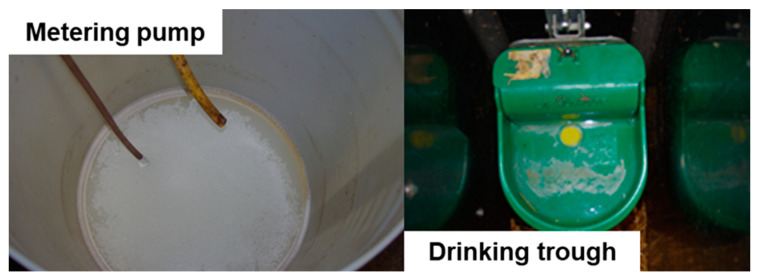
Deposit found in the metering pump and in the drinking troughs on the 4th and last day of treatment of lambs via the drinking water with an SDM-TMP combination (Trisulmix^®^ Liquid, 37.4 mg/kg/24 h SDM + 8.0 mg/kg/24 h TMP). The metering pump was set at 4%. The theoretical concentrations in the metering pump and in the drinking troughs during treatment were 9.35 g/L for SDM + 2 g/L for TMP and 374 mg/L for SDM + 80 mg/L for TMP, respectively.

On the first treatment day, the solution in the metering pump was apparently homogeneous and no deposit was observed in the drinking troughs. However, a deposit gradually accumulated over the next three days of treatment. The deposit found in the metering pump and drinking troughs on the 4th and last day of treatment is shown in Scheme 1. The SDM and TMP concentrations in the metering pump were calculated so that the lambs received 37.4 mg/kg BW/24 h of SDM and 8.0 mg/kg BW/24 h of TMP, according to a theoretical water intake of 2.5 L per animal and per day, with the metering pump set at 4%. According to these settings, the theoretical concentrations of SDM and TMP in the troughs should be equal to 374 and 80 µg/mL, respectively. The SDM concentrations in the drinking troughs increased from the 1st to 3rd day of treatment, becoming more stable and close to 100% of the theoretical concentration on the 3rd and 4th day of treatment (Table 3). After the end of treatment, SDM quickly disappeared from the watering system. The TMP concentrations recorded in the drinking troughs were well below the desired concentration with a maximum of 25.7 ± 15.9% of the theoretical concentration on the 4th day of treatment. Besides, 3 days after the end of treatment, the TMP concentrations in the troughs remained similar to the concentrations obtained during the treatment.

#### 2.4.2. Second Field Experiment with SDM Alone

**Scheme 2:**
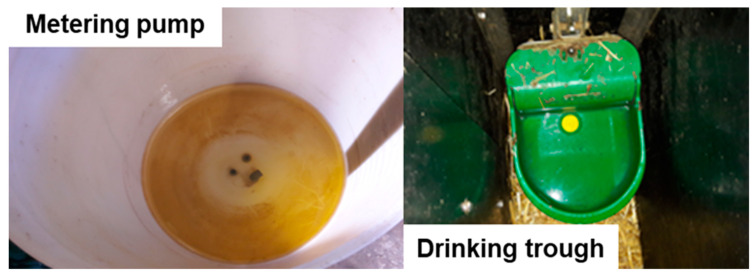
Deposit found in the metering pump and in the drinking troughs on the 5th and last day of treatment of lambs via the drinking water with SDM (Emericid^®^ Sulfadimethoxine, 55.68 mg/kg for the first day and 27.84 mg/kg/24 h from the 2nd to the 5th day). The metering pump was set at 10%. The average SDM observed concentrations in the metering pump during the five treatment days were 4.83 g/L for the first day of treatment and 2.73 g/L for the other 4 days. The average SDM theoretical concentrations in the drinking troughs during the five treatment days were 483 µg/mL for the first day of treatment and 273 µg/mL for the other 4 days.

For all treatment days, the solution in the metering pump appeared homogeneous and no deposit was observed in the drinking troughs (Scheme 2). The SDM concentrations in the metering pump were calculated so that the lambs received 55.68 mg/kg BW of SDM on the 1st day and 27.84 mg/kg BW/24 h of SDM for the 4 next days, according to a theoretical water intake of 2.5 L per animal and per day, with the metering pump set at 10%. By using these settings and considering the effective daily concentrations in the metering pump, the theoretical concentrations of SDM in the troughs should be equal to 483, 299, 289, 268 and 238 µg/mL for the 1st to 5th days of treatment, respectively. The SDM concentrations in the drinking troughs were stable and close to 100% of the theoretical concentration from the 1st day of treatment (Table 4). No SDM was found in the drinking troughs when the treatment was stopped and the pipes were flushed.

### 2.5. Individual Pharmacokinetics of SDM and TMP Administered in Combination via the Drinking Water

**Figure 5:**
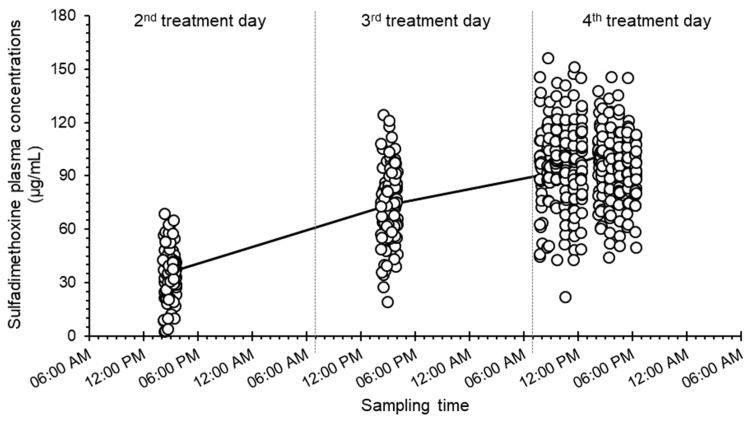
Individual SDM plasma concentrations for the 100 lambs in pen 2 that received an SDM-TMP combination (Trisulmix^®^ Liquid, 37.4 mg/kg/24 h SDM + 8.0 mg/kg/24 h TMP) for 4 days in the drinking water. The stock solution in the metering pump was renewed every morning around 07:00 am. Each circle corresponds to the plasma concentration for one lamb. The solid line links the average plasma concentrations.

The TMP plasma concentrations were below the LOQ of 1 µg/mL for all 100 lambs regardless of the day of collection. The SDM plasma concentrations are presented in Figure 5. They were between 2.61 and 68.90 µg/mL (35.8 ± 14.2 µg/mL) on the 2nd day of treatment, between 19.35 and 124.45 µg/mL (73.9 ± 21.9 µg/mL) on the 3rd day of treatment and between 21.94 and 156.15 µg/mL (95.0 ± 22.6 µg/mL) on the 4th day of treatment, demonstrating that the average SDM concentrations were nearly 3 fold higher on the 4th day than on the 2nd day of treatment.

### 2.6. Individual Pharmacokinetics of SDM Administered Alone via the Drinking Water

To limit the time required for SDM plasma concentrations to reach steady-state, in the second field experiment we planned to start with a loading dose of 55.68 mg/kg BW SDM on the 1st day followed by a dose of 27.84 mg/kg BW/24 h SDM from the 2nd to the 5th day. Since TMP actually hardly reached the troughs and seemed to precipitate, a formulation that only contained SDM (Emericid^®^ Sulfadimethoxine, Virbac, Carros, France) was selected for this experiment.

**Figure 6:**
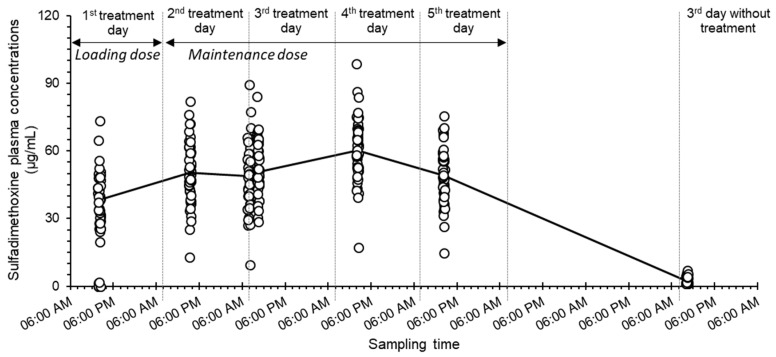
Individual SDM plasma concentrations for lambs (n = 100) that received a SDM treatment (Emericid^®^ Sulfadimethoxine, 55.68 mg/kg for the first day and 27.84 mg/kg/24 h from the 2nd to the 5th day) for 5 days in the drinking water. Each circle corresponds to the plasma concentration for one lamb. On the third day after the last treatment day, the SDM plasma concentrations were above the LOQ (Limit of Quantification) of 1 µg/mL for 36 out of 50 lambs. The solid line links the average plasma concentrations.

The individual SDM plasma concentrations obtained in the lambs are presented in Figure 6. They were similar for the 5 days of treatment and ranged from 1.28 to 73.24 µg/mL on the 1st day of treatment (38.1 ± 13.8 µg/mL), from 12.97 to 81.94 µg/mL (50.2 ± 13.6 µg/mL) on the 2nd day of treatment, from 9.35 to 89.22 µg/mL (49.8 ± 13.2 µg/mL) on the 3rd day of treatment, from 17.05 to 98.49 µg/mL (60.2 ± 13.8 µg/mL) on the 4th day of treatment, and from 14.84 to 75.51 µg/mL (49.0 ± 12.0 µg/mL) for the 5th day of treatment.

## 3. Discussion

Although several products containing a combination of sulfonamide and TMP have been registered for oral route in sheep, little if any information is available on their pharmacokinetic properties, especially when administered via the drinking water, which implies that the blood exposure of animals to the drugs after such treatments is uncertain. To promote a rational use of antibiotics and avoid useless antibiotic consumption, a better knowledge is required of the animal’s blood exposure to drugs after treatments via the drinking water.

The first issue with using drinking water to deliver a drug is the drug’s solubility which needs to be very high, considering the very concentrated stock solution required upstream of the metering pump. For many drugs/formulations, no information is available about solubility at the time of registration, even though precipitation can occur in water especially at low temperature, extreme (mainly alkaline) pH, or in hard water. In this study, the low in vitro solubility of one of the drugs, even after addition of a diluent, excluded it from further experiments. A recent review on water medication reported that this problem of solubility in the stock solution could also occur for other antimicrobial drugs [18]. In our study, one product was soluble at high concentrations for 2 h at +4 °C in the laboratory and the solution seemed homogeneous in the metering pump with no deposit on the first treatment day but eventually a deposit appeared both in the pump and the drinking troughs as the days went by. These observations demonstrate that an apparent solubility at the time of stock solution preparation may not be sufficient to ensure solubility in the pipelines throughout the treatment period. Stability of the drug in the stock solutions is another issue because these solutions are usually prepared once or at most twice a day in farms. Here, SDM seemed to remain stable since the expected concentration was found in the troughs at all sampling times during the treatment with SDM alone.

The need to obtain drug solubility and stability data before administering a new treatment in drinking water is therefore paramount. However, to comply with EMA recommendations, relatively old drugs (sulfonamides, tetracyclines, penicillins, etc.), for which solubility and pharmacokinetics data are usually poor, are preferentially selected for oral medication.

To determine the exposure of animals to SDM-TMP, we first examined the plasma concentration profiles obtained after individual administration of the SDM-TMP combination (Trisulmix^®^ Liquid for oral administration and Trisulmix^®^ Injectable for intravenous administration) in experimental settings. The dose ratio of SDM:TMP in these formulations is 5:1, which is the most common. This ratio was first established in human medicine to ensure the greatest synergistic effect on bacteria and the ratio of peak plasma concentrations of sulfamethoxazole:TMP in human patients was thus 20:1 [19,20,21]. To be able to use this same dose ratio in veterinary medicine, the pharmacokinetic profiles of the drugs would need to be similar in animals and humans to preserve the synergistic effect. However, several studies have shown that TMP pharmacokinetics are highly dependent on the species examined. For example, the half-life of TMP is 10–14 h in humans [22], whereas it is about 35–44 min in sheep [9,10]. We also found a similar half-life of 0.47 ± 0.23 h after IV administration. Indeed, TMP is supposed to be extensively metabolized by the liver in cattle and goats [23]. Being a weak base, TMP can also be trapped in tissues and in the rumen where it can then be degraded by the local microflora. The same mechanisms could also explain the very low bioavailability already observed after oral route in ruminants [17,23] and confirmed here in experimental settings. Thus, the very short half-life of TMP suggests that the contribution of TMP as a potentiator of SDM would be very poor in sheep, whatever the route of administration, and the very low oral bioavailability reinforces this drawback when the drug is given orally. In addition, our results indicate that the presence of TMP in the SDM-TMP formulation could decrease the solubility of the product. Indeed, deposits were observed in the pipelines, the expected concentrations of TMP were never attained in the troughs and, more importantly, residual TMP concentrations were observed for several days after the end of treatment in the troughs. This sustained presence of TMP in the ducts could exert selective pressure on the bacterial biofilms formed in the pipelines and favor the selection of antimicrobial resistance in the farm environment. Although SDM exhibited a far better behavior as it rapidly attained the expected concentrations in the troughs and rapidly disappeared from the pipes at the end of the treatment, these concentrations were much more variable when SDM was combined with TMP than when administered alone.

Different pharmacokinetic profiles after oral administration have been reported for sulfonamides in ruminants. Some sulfonamides such as sulfadimidine (=sulfamethazine), sulfanilamide or sulfamerazine have low bioavailabilities by oral route whereas others such as sulfamethoxazole, sulfatroxazole, or sulfadiazine have oral bioavailabilities exceeding 70% in adult goats or calves [14,15,17]. Previous studies have also shown that age and diet can affect the disposition of sulfonamides with, for example, a slight decrease in bioavailability observed in animals fed with grain as compared to animals receiving milk [17]. In this study, the bioavailability of SDM by oral route was complete for both pre-ruminant and ruminant lambs, implying that the oral route would be suitable for lambs of any age. SDM is reported to have a long half-life in many species, with a half-life of 12.5 h in cattle [12]. In our study, a similar terminal half-life was found in lambs with averages of 21.0 and 17.3 h after administration by oral and intravenous routes, respectively. This long terminal half-life can decrease the intra-individual variability of SDM concentrations but can also increase the time required to attain the steady-state concentrations [13]. Considering a half-life of 17 h, an administration once a day and equation 5 [13], the plasma concentrations of SDM at steady state should have been 1.6-fold higher than the concentrations observed on the 1st day of treatment. Under our conditions, we found a greater difference between the 2nd and 4th days of treatment, the average plasma concentrations in lambs being 2.7-fold higher on the 4th day than on the 2nd day. One explanation could be that the SDM concentrations in drinking troughs were lower than expected on the first days of treatment with the SDM-TMP combination. Another explanation could be that the lambs delayed water consumption and thus antibiotic intake on the 1st day of treatment. Low exposure to SDM during the first days of treatment is not desirable, because efficacy against the pathogens might be delayed and allow the spread of the disease over a longer period. Moreover, underexposure to antibiotics, while being useless to control the pathogens, can promote the selection of resistance. To address this issue of low concentrations at the beginning of treatment, we first tried to improve the supply of SDM to the drinking troughs. As TMP concentrations in drinking troughs were very low, we suspected that TMP precipitates and could at the same time lower the SDM solubility. We thus decided to administer SDM alone, expecting the subsequent solubility and concentrations in the drinking troughs to be closer to those required from the very beginning of the treatment. As the terminal half-life of SDM was quite long, we therefore planned a loading dose (twice the maintenance dose) for the 1st day of treatment. This new dosing regimen led to very similar average plasma concentrations of 38.1 (1st day), 50.2 (2nd day), 49.8 (3rd day), 60.2 (4th day) and 49.0 (5th day) µg/mL, with a 1.3-fold difference between the 1st and 5th day at the population level.

The individual plasma concentrations of SDM in lambs ranged from 1.28 to 98.49 µg/mL (49.8 ± 14.6 µg/mL) during the 5-day treatment. Such high intra- and inter-individual variabilities can result in ineffective treatments or toxicity in some animals with extremely low or high exposures. The measurements of water consumption did not reveal any decrease in consumption in the pens at the start of treatment suggesting that the taste of the drug was accepted by the animals. Individual water consumption by pigs is reported to be influenced by numerous factors including stress, boredom, environmental temperature, disease, feed type and constituents and water flow rates [18], but in our study, despite the same age, weight, environment, food and health status, the individual consumption varied considerably between the lambs. Soraci et al. [6] showed that consumption in healthy animals was also dependent on social rank, even if the effect was lower for water than for feed consumption. Here, the estimated individual daily drinking volumes ranged from 0 to 9.8 L implying that the individual daily doses, for a SDM concentration in the drinking troughs of 374 µg/mL, ranged from 0 to 147 mg/kg BW while the targeted one was 37.4 mg/kg BW. These different doses probably explain most of the observed inter-individual variability in plasma concentrations but the time-development of drinking behavior of each lamb, with either frequent or infrequent visits to the troughs, could also accentuate this variability. In pigs, one or two peaks of water consumption were observed over each 24-h period [18]. We also observed a similar trend with two peaks, mainly during daytime, in the lamb population. However, at the individual level, some lambs behaved very differently, drinking frequently throughout the day for no obvious reason. Under epidemic conditions, the variability between animals can be even higher due to a potential influence of the disease on drinking behavior and additional studies should be carried out to check if sick animals are at least as exposed to drugs as healthy animals. Success of the treatment at the population level will then rely on attaining a defined target value for the relevant pharmacokinetic/pharmacodynamic index, which is dependent both on exposure to the drug and on the MIC of pathogens, in a sufficiently high proportion of the animals within the group. Here, we showed that the synergy of SDM and TMP was lost in sheep, due to the absence of TMP in sheep plasma, and that the efficacy of SDM alone to control pathogenic bacteria should therefore be considered. Unfortunately, most of the published susceptibility data on respiratory pathogens such as *Mannheimia haemolytica* and *Pasteurella multocida* are provided for the sulfamethoxazole/TMP combination [24] and very few data are available for sulfonamides alone. In any case, even if adequate exposure to the drug at the population level would control disease spread within the herd, a second-line treatment would be required for a few animals due to unavoidable individual underexposure to the drug.

## 4. Materials and Methods

### 4.1. Solubility in Water of Formulations Containing the SDM-TMP Combination or SDM Alone

Solubility in drinking water of two different formulations of SDM-TMP (Trisulmix^®^ Powder and Trisulmix^®^ Liquid, Coophavet, Ancenis, France) and one formulation of SDM alone (Emericid^®^ Sulfadimethoxine, Virbac, Carros, France) was assessed in water with different combinations of pH (5.5, 6.5, 7.5 or 8.5) and hardness (10 °f, 30 °f and 50 °f), representative of those found in sheep fattening houses. A carbonate buffer was first prepared, the pH was then adjusted with HCl 2 M or NaOH 10 M and the hardness with CaCO_3_ to create the different testing conditions.

Based on the labelled doses of 30 mg/kg BW SDM and 6.5 mg/kg BW TMP for Trisulmix^®^ Powder and Trisulmix^®^ Liquid, the predicted water consumption per animal (1 L/10 kg BW/day) and the pump dilution rate of 10%, the maximum concentration calculated for the different formulation in the metering pump were 17 g of Trisulmix^®^ Powder and 17 mL of Trisulmix^®^ Liquid per liter of water. Based on the labelled dose of 55.68 mg/kg BW for Emericid^®^ Sulfadimethoxine, the predicted water consumption per animal (1 L/10 kg BW/day) and the pump dilution rate of 6%, the maximum concentration calculated for the formulation in the metering pump was 43.2 mL of Emericid^®^ per liter of water.

The solubility of the different formulations was first tested after 2 h at room temperature (RT), then at +4 °C if the product was soluble at RT or at +30 °C if the product was not soluble at RT. For formulations containing the SDM-TMP combination, the experiments were carried out in the presence or not of an organic diluent (Super Diluant Virbac^®^, Virbac, Carros, France) which is sometimes recommended to increase drug solubility in the dosing pump in sheep fattening houses. For all the tests, the level of product solubility was determined visually. The most soluble formulations were used to determine the pharmacokinetic parameters of SDM and TMP in experimental settings and for the treatment of animals in fattening houses.

### 4.2. Animals

All the lambs were Lacaune or Lacaune crossbreds supplied by the agricultural cooperative Arterris (Castelnaudary, France). The experimental protocols were authorized by the French Ministry of Research under the number #4637_2016032217062253 on 11 May 2017 for the laboratory experiment done at the INTHERES animal facility and the number #11919_2017102415533573 on 26 June 2018 for the two experiments conducted in a sheep fattening house managed by the agricultural cooperative Arterris.

### 4.3. Pharmacokinetic Parameters of SDM and TMP in Experimental Settings

Eight, 1-month-old lambs (4 males and 4 females) were weaned on the day of their arrival in the animal facility as they would have been on entering the fattening house. They were fed ad libitum with a starter feed for 10 days and then with a maintenance feed, these feeds being the same as those used in the fattening house and free of antibiotics. The lambs also had free access to water and straw. They received one dose of diclazuril on arrival and another one 10 days later (Vecoxan^®^ 2.5 mg/mL, 1 mg/kg BW, Elanco, Sèvres, France) to reduce the risks of coccidiosis. Six days after their arrival and weaning, the 8 lambs (13.5 ± 1.8 kg BW) received an oral bolus of SDM-TMP (Trisulmix^®^ Liquid, 24.7 mg/kg BW SDM + 5.3 mg kg BW TMP). Blood samples were collected just before drug administration and 0.25, 0.75, 1.5, 2, 2.5, 4, 6, 9, 24, 30, 48 and 72 h after administration. Seven days later, the lambs (15.1 ± 2.1 kg BW) received an intravenous bolus of SDM-TMP (Trisulmix^®^ Injectable, 24.7 mg/kg BW SDM + 5.3 mg/kg BW TMP, Coophavet, Ancenis, France). Blood samples were collected just before administration and 0.08, 0.25, 0.5, 1, 2, 4, 6, 9, 24, 30, 48 and 72 h after administration, to determine the oral bioavailability of SDM and TMP. Finally, again seven days later (that is 14 days after the first oral administration and 20 days after weaning), the oral administration was repeated on the same lambs (17.6 ± 2.8 kg BW) to determine whether the pharmacokinetics of SDM and TMP could be influenced by the lamb’s ruminal status. Each 2 mL blood sample was taken from one jugular vein (for the intravenous administration, jugular opposed to the one used for the administration) and collected in heparinized tubes. The samples were then centrifuged at 3000× *g* for 10 min at +4 °C, and the collected plasma stored at −20 °C before assay.

### 4.4. Individual Pharmacokinetics of SDM and TMP in Combination and Individual Water Consumption in Fattening Houses

Two hundred lambs, around 40 days old, were dosed with Trisulmix^®^ Liquid (37.4 mg/kg BW/24 h SDM + 8.0 mg/kg BW/24 h TMP) for 4 days via drinking water. A sufficient volume of stock solution was prepared once a day in the metering pump to supply the drug in the pipelines over 24 h. The metering pump was set at 4%. Dissolution of the SDM-TMP was facilitated by adding Super Diluant Virbac^®^ to the metering pump (Trisulmix^®^ Liquid:Super Diluant Virbac^®^, 1:1). The treated lambs were allocated to two pens of 100 lambs (50 males and 50 females), each pen being equipped with two constant-level drinking troughs. The lambs in pen 1 were neither sampled nor handled, in order to assess water consumption before, during and after treatment with limited human influence, while the lambs in pen 2 were sampled several times to quantify the plasma SDM and TMP concentrations. The individual water consumption was determined in real time for all the lambs in pens 1 and 2 from three days before treatment to three days after the end of treatment using water meters connected to the drinking troughs which detected the RFID chip in the lamb ear tags. Lamb access to the drinking troughs was adapted to allow only one lamb at a time. Water samples were taken throughout the duration of treatment from the two drinking troughs in each of the two pens (n = 4) before, and 1 h, 4 h (except on the 4th day), 8 h and 12 h (except on the 3rd day) after the renewal of the treatment in the metering pump each morning, and from one drinking trough in each of the two pens once per day for 4 days after the treatment. The water samples were collected in vials stored at +4 °C until the assays. The lambs in pen 2 were divided into 4 batches of 25 lambs. One blood sample per lamb was taken on the 2nd and 3rd days of treatment and four blood samples per lamb, taken between 07:30 and 18:30 at 12 sampling times with 25 sampled lambs at each time, were obtained on the 4th day of treatment. The blood samples were collected in heparinized tubes, centrifuged at 3000× *g* for 10 min at +4 °C, and the collected plasma stored at −20 °C.

### 4.5. Individual Pharmacokinetics of SDM Alone in Fattening Houses

The number and characteristics of the treated animals were identical to those in the first experiment. This time, the 200 lambs were dosed with Emericid^®^ Sulfadimethoxine (55.68 mg/kg BW SDM on the 1st day and 27.84 mg/kg BW/24 h SDM from the 2nd to 5th day) for 5 days via the drinking water. A sufficient volume of stock solution was prepared once a day in the metering pump to supply the drug in the pipelines over 24 h. The pump was set at 10%. Water samples were taken throughout the duration of treatment: from the metering pump before and 3 h after renewal of the treatment, from the drinking troughs several times during the treatment, twice on the day the treatment was stopped and once every two days for 5 days after the treatment. One blood sample was obtained from the lambs of batch 1 (n = 50) in pen 2 on the 1st, 3rd and 4th days of treatment and the 3rd day (Day 8) after the end of treatment. One blood sample was obtained from the lambs of batch 2 (n = 50) in pen 2 on the 2nd, 3rd and 5th days of treatment and the 5th day (Day 10) after the end of treatment. The plasma and water samples were processed as previously described.

### 4.6. SDM and TMP Assays

#### 4.6.1. SDM and TMP Assays in Plasma in the Laboratory Experiment

SDM and TMP plasma concentrations were determined by LC/MS with an Acquity ultra performance liquid chromatography (UPLC^®^) coupled to a Xevo^®^ triple quadrupole mass spectrometer (Waters, Milford, MA, USA). Plasma samples (50 µL) were spiked with 150 µL of IS (Internal Standard) sulfapyridine at 0.1 µg/mL in trichloracetic acid (TCA 5%) and centrifuged for 10 min, at 20,000× *g* and +4 °C. The analytes were separated on a C18 column (Cortecs UPLC C18+, 2.1 × 50 mm, 1.6 µm, Waters) with an H2O, 0.1% HCOOH/AcN gradient elution (t(0 min): 10% AcN, t(0–2 min): 60% AcN ; t(2–2.10 min): 10% AcN ; t(2.10–3 min): 10% AcN). Samples were detected by multiple reactions monitoring (MRM) with a positive electrospray ionization. The MRM transitions monitored were m/z: 250 > 156, m/z: 291 > 230 and m/z: 311 > 108 for sulfapyridine, TMP and SDM, respectively, with collision energies of 16, 24 and 32 eV. The retention times were 1.38, 0.79 and 0.76 for SDM, TMP and IS, respectively. The performance of the method was checked in terms of linearity, inter- and intra-day precision and accuracy, and sensitivity. Six calibration points containing SDM and TMP at concentrations ranging from 0.01 to 5 µg/mL for TMP and from 0.25 to 50 µg/mL for SDM were extracted and assayed over three days. Both linear (Y = aX + b) and quadratic (Y = aX^2^ + bX + c) models were tested with 1, 1/X and 1/X^2^ (X = nominal concentration) weightings with these resulting calibration curves. Three approaches were evaluated to select the best model of calibration: (1) the inspection of the residual distribution plotting against nominal concentrations, (2) the lack-of-fit test to check the goodness-of-fit of the model, and (3) the calculation of the relative concentration residuals (RCR%) between the nominal concentration and the concentration obtained with the model, which should be lower than ± 15%. The best calibration fit was obtained with a quadratic model weighted by 1/X^2^ (X = concentration) for both molecules with RCR% were lower than 15% for all concentrations. The LOQ (limit of quantification) was evaluated with five replicates of plasma samples spiked at 0.25 µg/mL for SDM and 0.01 µg/mL for TMP. They were set as the lowest concentration level of the calibration curve that can be quantified with an acceptable repeatability and accuracy (CV% lower than 20% and accuracy range 80–120%). The accuracies and the intra- and inter-day precisions of the method were evaluated with five replicates of three quality control (QC) samples at three concentration levels (0.025, 0.25 and 2.5 µg/mL for TMP and 0.25, 2.5 and 25 µg/mL for SDM) over three days. Intra and inter-day precision were expressed with coefficient of variation percent (CV%) and calculated with an ANOVA. The intra-day CV% precision was below 11% and 8% and the inter-day CV% precisions were below 19% and 18% for TMP and SDM, respectively. The accuracies ranged from 104% to 121% for TMP, and from 81% to 93% for SDM.

#### 4.6.2. SDM and TMP Assays in Plasma and Drinking Water in the Field Experiments

As a very low level of TMP was detected in the plasma with the previous method and during the previous experiment, and SDM response in the field experiment saturated the MS signal with the previous method, a LC/UV method was developed solely for SDM in plasma. This method using higher concentration levels was also adapted to quantify TMP and SDM in water.

Briefly, SDM and TMP were determined by LC/UV with an Acquity ultra performance liquid chromatography (UPLC^®^) coupled to a diode array detector (Waters, Milford, MA, USA). The analytes were detected at 270 nm and were eluted under the same conditions as described for the laboratory experiment. SDM was extracted from plasma (100 µL) with 300 µL of IS sulfapyridine at 10 µg/mL diluted in TCA 5% and centrifuged for 10 min, at 20,000× *g* and +4 °C. The performance of the method was evaluated with a calibration ranging from 1 to 500 µg/mL using a linear model weighted by 1/X and three QC samples (3, 30 and 300 µg/mL). The accuracy ranged from 83% to 104% and intra-day CV% precision was below 13% and inter-day CV% precision was below 14%. The LOQ was set at 1 µg/mL with an intra-day CV precision of 6% and an accuracy of 106%. In water, 100 µL of samples were directly diluted with 300 µL of TCA 5%. As the run took only 3 min, all samples were processed on the same day. The calibration curve ranged from 5 to 1000 µg/mL and from 0.5 to 100 µg/mL for SDM and TMP, respectively.

### 4.7. Pharmacokinetic and Statistical Analyses

For the laboratory experiment, the non-compartmental analysis was conducted with Phoenix^®^ software (Phoenix, WinNonlin 64, NLME 1.6, Certara L. P., Pharsight, St-Louis, MO, USA). As (i) the clearance after the intravenous administration was higher than the apparent clearance after the oral administrations and (ii) we cannot exclude a carryover effect between the different administrations, the bioavailabilities by oral route were corrected by addition of the terminal half-life term for each route of administration in the calculation [25]. The bioavailabilities by oral route were thus determined with Equation (1):

(1) $$F\;=\;\frac{{\mathrm{AUC}}_{\mathrm{oral}}}{{\mathrm{AUC}}_{\mathrm{iv}}}\;\times \;\frac{t_{1/2,\mathrm{iv}}}{t_{1/2,\mathrm{oral}}}\;\times \;\frac{{\mathrm{Dose}}_{\mathrm{iv}}}{{\mathrm{Dose}}_{\mathrm{oral}}}$$

where AUC_oral_ and AUC_iv_ are the Area Under Curve from time 0 extrapolated to infinite time after oral and intravenous administrations, t_1/2, iv_ and t_1/2, oral_ are the terminal half-life after intravenous and oral administrations, and Dose_iv_ and Dose_oral_ are the actual dose administered by intravenous and oral route.

The influence of ruminal status on the SDM pharmacokinetic parameters after an oral administration was analyzed by a non-parametric test (Wilcoxon test) with R^®^ software (R 3.4.3, R Development Core Team, Vienna, Austria).

## 5. Conclusions

In conclusion, we showed that, beyond this example of sulfonamides and TMP, medication via the drinking water will require investigation of a drug’s solubility and pharmacokinetics as well as animal behavior to avoid inadequate exposure at the population level and to remain compliant with a rational use of antibiotics in veterinary medicine.

## Figures and Tables

**Table 1:** Pharmacokinetic parameters of SDM (mean ± SD) after single administrations of a SDM-TMP combination orally (Trisulmix^®^ Liquid, 24.7 mg/kg SDM + 5.3 mg/kg TMP) or intravenously (Trisulmix^®^ Injectable, 24.7 mg/kg SDM + 5.3 mg/kg TMP) to eight lambs.

Pharmacokinetic Parameters	Oral Administration 1 «Pre-ruminant» Status	Intravenous Administration	Oral Administration 2 «Ruminant» Status
C_max_obs_ (µg/mL)	16.00 ± 9.86	-	17.81 ± 6.20
T_max_ (h)	5.14 ± 2.31	-	4.69 ± 2.83
AUC_0-inf_ (µg.h/mL)	489.8 ± 169.7	282.9 ± 99.8	417.1 ± 116.2
Cl or Cl/F (mL/min/kg)	0.75 ± 0.28	1.12 ± 0.35	0.88 ± 0.23
t_1/2_ (h)	24.24 ± 4.36	17.26 ± 2.25	17.77 ± 5.36 *
Vss or Vss/F (L/kg)	1.57 ± 0.62	1.72 ± 0.61	1.33 ± 0.49
F	1.09 ± 0.23	-	1.25 ± 0.21

C_max_obs_ = observed peak plasma SDM concentration, T_max_ = time at which the C_max_ is reached, AUC_0-inf_ = Area Under Curve from time 0 extrapolated to infinite time, Cl = clearance and Cl/F = apparent clearance, t_1/2_ = terminal half-life, Vss = volume of distribution at steady-state and Vss/F = apparent volume of distribution at steady-state, F = bioavailability. Significantly different between the two oral administrations: * *p* < 0.05.

**Table 2:** Pharmacokinetic parameters of TMP (mean ± SD) after a single administration of a SDM-TMP combination intravenously (Trisulmix^®^ Injectable, 24.7 mg/kg SDM + 5.3 mg/kg TMP) to eight lambs.

Pharmacokinetic Parameters	Intravenous Administration
AUC_0-inf_ (ng.h/mL)	644.0 ± 157.2
Cl (mL/min/kg)	178.7 ± 36.05
t_1/2_ (h)	0.47 ± 0.23
Vss (L/kg)	5.58 ± 2.34

AUC_0-inf_ = Area Under Curve from time 0 extrapolated to infinite time, Cl = clearance and Cl/F = apparent clearance, t_1/2_ = terminal half-life, Vss = volume of distribution at steady-state. The pharmacokinetic parameters of TMP after a single oral administration of an SDM-TMP combination (Trisulmix^®^ Liquid) could not be estimated.

**Table 3:** Percentage of the theoretical concentrations of SDM and TMP (mean ± SD and [range]) found in the drinking troughs before, during and after the SDM-TMP treatment of 200 lambs for 4 days via the drinking water (Trisulmix^®^ Liquid, 37.4 mg/kg/24 h SDM + 8.0 mg/kg/24 h TMP). Before and during the SDM-TMP treatment, water was collected from all the drinking troughs (n = 4, two in two different pens). Just before treatment end and after the SDM-TMP treatment, water was collected from one drinking trough/pen (n = 2) and the two obtained values are reported separately.

During Treatment	Sampling Time	Mean (± SD)% [Min–Max] of the Theoretical Concentrations SDM Theoretical Concentration = 374 µg/mL TMP Theoretical Concentration = 80 µg/mL	
		SDM	TMP
1st day of treatment	Before treatment T_1_	-	-
	T_1_ + 1 h	2.0 ± 2.2% [0.8–5.4]	2.2 ± 1.4% [1.0–4.1]
	T_1_ + 4 h	15.7 ± 13.6% [0.5–33.4]	5.7 ± 5.2% [0.7–12.1]
	T_1_ + 8 h	50.5 ± 21.3% [23.2–74.5]	16.8 ± 8.3% [11.2–29.1]
	T_1_ + 12 h	**92.1 ± 7.5%** [81.1–97.8]	5.7 ± 2.1% [3.6–8.5]
2nd day of treatment	Before treatment T_2_	27.2 ± 20.6% [9.7–56.7]	10.5 ± 3.8% [5.4–14.4]
	T_2_ + 1 h	55.2 ± 24.6% [29.5–83.8]	15.2 ± 9.1% [6.7–25.5]
	T_2_ + 4 h	84.2 ± 3.7% [78.8–87.2]	8.8 ± 2.8% [6.0–12.3]
	T_2_ + 8 h	**100.9 ± 2.5%** [98.6–103.7]	6.6 ± 0.6% [5.8–7.3]
	T_2_ + 12 h	76.4 ± 16.5% [59.4–97.5]	9.4 ± 2.7% [5.4–11.1]
3rd day of treatment	Before treatment T_3_	24.2 ± 16.4% [11.7–46.3]	13.2 ± 4.0% [8.3–17.6]
	T_3_ + 1 h	**124.7 ± 42.4%** [65.3–158.0]	4.5 ± 3.1% [1.7–8.9]
	T_3_ + 4 h	**106.7 ± 31.5%** [70.9–145.7]	5.0 ± 2.9% [2.6–9.2]
	T_3_ + 8 h	**118.9 ± 33.6%** [76.2–158.0]	4.2 ± 1.6% [2.9–6.3]
4th day of treatment	Before treatment T_4_	**125.6 ± 12.4%** [110.5–137.8]	3.3 ± 1.0% [2.0–4.3]
	T_4_ + 1 h	79.0 ± 19.7% [61.6–96.3]	9.2 ± 6.1% [4.7–17.7]
	T_4_ + 8 h	**94.7 ± 1.2%** [93.5–95.7]	5.3 ± 0.9% [4.6–6.6]
	T_4_ + 12 h	67.2 ± 18.8% [44.0–82.5]	25.7 ± 15.9% [5.6–43.7]
Before treatment end	T_4_ + 24 h	66.3/66.6%	10.3/29.6%
**After treatment**	**Sampling time**	Mean (±SD)% of theoretical concentrations remaining in the drinking troughs after treatment/theoretical concentrations in the drinking troughs before treatment end *	
1 day after	-	1.2/2.2%	10.8/31.9%
2 days after	-	7.5/8.4%	69.7/182.7%
3 days after	-	6.0/6.9%	51.7/180.7%

The theoretical concentrations in the drinking troughs were estimated for a metering pump set at 4% and a theoretical concentration in the metering pump of 9.35 g/L for SDM and 2 g/L for TMP. The theoretical concentration in the metering pump was calculated with the following equation:
$$\begin{array}{l} \mathrm{Concentration}\;\mathrm{in}\;\mathrm{the}\;\mathrm{metering}\;\mathrm{pump}\;\left(\mathrm{mg}/L\right)\\ =\frac{\mathrm{Dose}\;\left(\mathrm{mg}/\mathrm{kg}/\mathrm{day}\right)\times \mathrm{Average}\;\mathrm{lamb}\;\mathrm{weight}\;\left(\mathrm{kg}\right)}{\mathrm{Average}\;\mathrm{water}\;\mathrm{consumption}\;\mathrm{per}\;\mathrm{lamb}\;\left(\frac{L}{\mathrm{day}}\right)\times \mathrm{pump}\;\mathrm{percentage}} \end{array}$$
with an average lamb weight of 25 kg, an average water consumption per lamb of 2.5 L/day and a pump dilution percentage of 0.04. The mean observed values above 90% are highlighted in bold. * After treatment, a percentage of the theoretical concentrations in the drinking troughs relative to the concentration in the metering pump could not be determined as the theoretical concentration in the metering pump was zero. Thus, the percentage expressed here represents the percentage of SDM or TMP remaining in the drinking troughs relative to the concentrations in the drinking troughs before the end of the treatment.

**Table 4:** Percentage of the theoretical concentrations of SDM found in drinking troughs during the SDM treatment of 200 lambs for 5 days via the drinking water (Emericid^®^ Sulfadimethoxine, 55.68 mg/kg SDM the 1st day and 27.84 mg/kg/24 h SDM from the 2nd to the 5th day). Depending on the sampling time, percentages were given as mean ± SD and [range] when water was collected from all the drinking troughs (n = 4, two in two different pens) and as two separate values when water was collected in only one drinking trough/pen (n = 2). No SDM was found in the drinking troughs as soon as the treatment was stopped and the pipes were flushed (not shown in the table).

During Treatment	Sampling Time	Number of Drinking Troughs Tested	Mean (±SD)% [Min–Max] of the Theoretical Concentrations
1st day of treatment	T_1_ + 1 h	n = 2, one per/pen	81.8/92.5%
	T_1_ + 3 h	n = 4, two per pen	**91.1 ± 10.9%** [74.9–98.3]
	T_1_ + 5 h	n = 2	86.7/93.2%
	T_1_ + 7 h	n = 4	**92.1 ± 1.5%** [90.2–93.8]
	T_1_ + 9 h	n = 2	89.9/95.6%
	T_1_ + 12 h	n = 4	**90.9 ± 3.0%** [87.3–94.7]
2nd day of treatment	Before treatment T_2_	n = 4	**98.4 ± 5.4%** [93.8–106.0]
	T_2_ + 1 h	n = 2	104.4 /115.3%
	T_2_ + 3 h	n = 4	88.8 ± 22.2% [55.7–103.5]
	T_2_ + 5 h	n = 2	98.7/102.3%
	T_2_ + 7 h	n = 4	**92.9 ± 6.5%** [86.4–101.3]
	T_2_ + 9 h	n = 2	99.3/100.6%
	T_2_ + 12 h	n = 4	**92.6 ± 6.6%** [83.1–97.2]
3rd day of treatment	Before treatment T_3_	n = 4	**97.3 ± 0.8%** [96.4–98.4]
	T_3_ + 1 h	n = 2	103.5/106.2%
	T_3_ + 3 h	n = 4	**103.5 ± 0.9%** [102.5–104.6]
	T_3_ + 5 h	n = 2	104.9/106.5%
	T_3_ + 7 h	n = 4	**92.3 ± 27.3%** [52.6–114.9]
	T_3_ + 9 h	n = 2	79.6/115.5%
4th day of treatment	Before treatment T_4_	n = 4	**110.0 ± 17.0%** [94.7–131.1]
	T_4_ + 1 h	n = 2	91.7/96.9%
	T_4_ + 3 h	n = 4	**95.7 ± 0.5%** [94.9–96.0]
	T_4_ + 5 h	n = 2	91.4/95.6%
	T_4_ + 7 h	n = 4	**92.3 ± 0.3%** [91.9–92.7]
	T_4_ + 9 h	n = 2	94.1/94.7%
	T_4_ + 12 h	n = 4	**92.2 ± 2.7%** [89.1–94.7]
5th day of treatment	Before treatment T_5_	n = 4	65.0 ± 37.2% [32.5–102.0]
	T_5_ + 1 h	n = 2	97.5/99.1%
	T_5_ + 3 h	n = 4	**92.1 ± 8.5%** [80.0–98.1]
	T_5_ + 5 h	n = 2	89.8/99.4%
	T_5_ + 7 h	n = 4	**98.0 ± 1.4%** [96.5–99.5]
	T_5_ + 9 h	n = 2	101.5/105.3%
	T_5_ + 12 h	n = 4	**98.0 ± 2.9%** [94.7–100.8]

The theoretical concentrations in the drinking troughs were estimated for a metering pump set at 10% and the observed concentrations in the metering pump for each treatment day. Then, the theoretical concentrations in the drinking troughs after each treatment renewal were estimated from the daily observed concentrations in the metering pump and were 483, 299, 289, 268 and 238 µg/mL from the 1st to 5th days of treatment, respectively. The mean observed values above 90% are highlighted in bold.
